# Histone chaperone HIRA facilitates transcription elongation to regulate insulin sensitivity and obesity-associated adipose expansion

**DOI:** 10.1101/2025.03.21.644577

**Published:** 2025-03-25

**Authors:** Danyang Wan, Ji-Eun Lee, Young-Kwon Park, Susanna Maisto, Christabelle Agyapong, Keiko Ozato, Oksana Gavrilova, Kai Ge

**Affiliations:** 1 Adipocyte Biology and Gene Regulation Section, National Institute of Diabetes and Digestive and Kidney Diseases, National Institutes of Health, Bethesda, MD 20892, USA; 2 Division of Developmental Biology, National Institute of Child Health and Human Development, National Institutes of Health, Bethesda, MD 20892, USA; 3 Mouse Metabolism Core, National Institute of Diabetes and Digestive and Kidney Diseases, National Institutes of Health, Bethesda, Maryland 20892, USA

## Abstract

Adipose tissue is essential for maintaining glucose and lipid homeostasis in mammals. The histone chaperone HIRA has been reported to play a lineage- and stage-selective role during development. However, its role in adipose tissue development and function as well as its working mechanism remain unknown. Here we show that tissue-specific knockout of histone chaperone HIRA in mice impairs insulin sensitivity and alleviates adipose tissue expansion during high-fat diet-induced obesity, but only moderately affects embryonic development of adipose tissue. Mechanistically, HIRA is selectively required for expression of genes critical for insulin response and lipogenesis, rather than adipogenesis, in adipose tissue. By acute depletion of HIRA protein and by mapping HIRA genomic localization in adipocytes, we demonstrate that HIRA binds to promoters and enhancers of insulin response and lipogenesis genes and regulates their expression by facilitating transcription elongation. Our findings not only identify HIRA as an epigenomic regulator of insulin sensitivity, lipogenesis, and obesity-associated adipose expansion, but also reveal a novel mechanism by which HIRA regulates transcription.

## Introduction

Adipose tissue plays an essential role in various physiological processes including energy storage, insulin sensitivity, and glucose and lipid homeostasis in mammals^1,2^. Adipose tissue is also considered an endocrine organ and secretes adipokines. Adiponectin is an adipokine specifically and abundantly expressed in adipose tissue. Development of adipose tissue (adipogenesis) is under the control of transcription factors (TFs) and epigenomic regulators^3^. The master adipogenic TF PPARγ cooperates with another key TF C/EBPα on transcriptional enhancers to induce expression of thousands of adipocyte genes^4^. Among them, genes encoding lipogenic TFs and enzymes are induced in the late phase of adipogenesis. Increases in adipocyte number due to adipogenesis (hyperplasia), and more importantly enlarged adipocytes (hypertrophy) due to lipogenesis or lipid uptake, lead to adipose tissue expansion in obesity^2,5^.

Adipose tissue and the liver are major lipogenic organs and coordinate the conversion of excess dietary carbohydrates into fatty acids through glycolysis, the tricarboxylic acid cycle, and de novo lipogenesis. Fatty acids are subsequently esterified into triglyceride for storage in lipid droplets ^6–8^. Glucose and insulin promote lipogenesis gene expression in liver and adipose tissue through activation of lipogenic TFs ChREBP and SREBP1^9,10^. ChREBP and SREBP1 directly promote expression of genes encoding lipogenesis enzymes ATP-citrate lyase (ACLY), acetyl-CoA carboxylase (ACACA), fatty acid synthase (FASN), and stearoyl-CoA desaturase 1 (SCD1), resulting in fatty acid synthesis and triglyceride accumulation in the late phase of adipogenesis^8^. Many epigenomic factors, including histone methyltransferases, histone acetyltransferases and chromatin remodeling enzymes, have been reported to positively or negatively regulate adipogenesis^3,8^. However, the role of histone chaperones in adipose tissue development, function and expansion has not been reported.

The histone chaperone HIRA (histone regulator A) deposits the histone H3 variant H3.3 into gene regulatory regions in a DNA synthesis-independent manner^11–15^. ChIP-Seq analyses in human HeLa and mouse embryonic stem (ES) cells revealed that HIRA colocalizes with H3.3 on promoters and active enhancers (AEs). Knockdown of *HIRA* expression markedly reduces H3.3 deposition at HIRA-positive enhancers and promoters ^16,17^. Depletion of HIRA does not affect ES cell self-renewal and only leads to mild changes in global gene expression, but impairs neural differentiation^12,17^. *Hira* KO mouse embryos show abnormal gastrulation and die before embryonic day E11^18^. HIRA is required for normal heart development and generation of all hematopoietic lineages in mice ^19,20^. Adult mice with muscle-specific deletion of *Hira* show impaired muscle regeneration^21^. These findings suggest a lineage- or stage-selective role of HIRA in differentiation and development.

In this study, we explored the role of HIRA in adipose tissue development, function and expansion. By crossing *Hira*^f/f^ with *Myf5-Cre* mice, we found that deletion of *Hira* in the embryonic stage only mildly affects brown adipose tissue (BAT) development but downregulates insulin response and lipogenesis genes. By crossing *Hira*^f/f^ with *Adipoq-Cre* mice, we observed that deletion of *Hira* in adipocytes renders mice resistant to adipose tissue expansion during high-fat diet (HFD)-induced obesity. RNA-Seq and ChIP-Seq analyses revealed that HIRA directly binds to promoters and enhancers of insulin response and lipogenesis genes to regulate their expression in adipocytes. Furthermore, we employed a rapid protein degradation system to assess, for the first time, the immediate effects of HIRA loss in adipocytes. Our data indicate that acute depletion of HIRA impairs transcription elongation on target genes.

## Results

### Deletion of *Hira* moderately affects BAT development but reduces expression of insulin response and lipogenesis genes

**Figure. 1.**
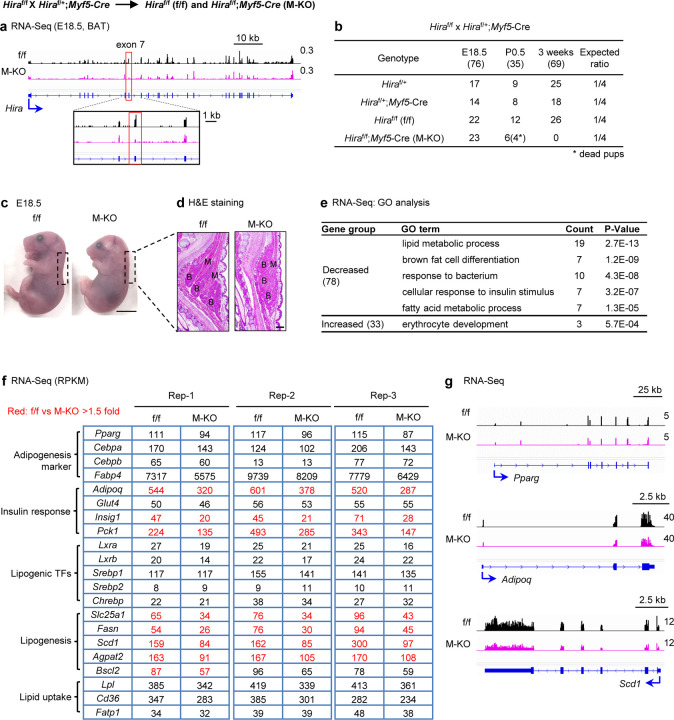
Deletion of *Hira* moderately affects BAT development but reduces expression of insulin response and lipogenesis genes **a**, Genome browser view of RNA-Seq data on the *Hira* gene locus. The targeted exon 7 is highlighted. **b**, Genotype of progeny from the crossing between *Hira*^f/f^ (f/f) and *Hira*^f/f^;*Myf5-Cre* (M-KO) mice at embryonic day 18.5 (E18.5), post-natal day 0.5 (P0.5) and 3 weeks of age. The asterisk indicates dead pups. **c**, Representative morphology of E18.5 embryos. Scale bar, 0.5 cm. **d**, H&E staining of E18.5 embryos. Sagittal sections of the cervical/thoracic area were stained with H&E. B, BAT; M, muscle. Scale bar, 0.5 mm. **e**, GO analysis of gene groups defined in Fig. 1-S1b-c. **f**, Expression levels of representative genes are shown in RPKM values of RNA-Seq data. g, RNA-Seq profiles on *Pparg*, *Adipoq* and *Scd1* loci in f/f and M-KO mice.

To study the role of HIRA in adipose tissue and muscle development, we crossed *Hira*^f/f^ (hereafter referred to as f/f) with *Myf5-Cre* mice to delete the *Hira* gene in progenitor cells of BAT and muscle lineages (Fig. 1-S1a)^8^. RNA-Seq analysis of interscapular BAT isolated from E18.5 embryos confirmed the deletion of *Hira* (Fig. 1a). *Hira*^f/f^;*Myf5-Cre* (hereafter referred to as M-KO) showed partial perinatal lethality and could not survive to the weaning age at 3 weeks (Fig.1b). M-KO embryos were obtained at the expected Mendelian ratio at E18.5 but displayed an abnormal hunched posture (Fig. 1b-c). Immunohistochemical analysis of cervical regions of E18.5 embryos revealed that deletion of *Hira* leads to a decrease of muscle mass, indicating that HIRA is required for muscle development (Fig. 1d). Consistently, Hira was required for myogenesis in C2C12 cells (Fig. 1-S1d-f). BAT sizes were moderately decreased in M-KO mice (Fig. 1d). RNA-Seq analysis of three biological replicates identified 78 and 33 genes down- or up-regulated over 1.5-fold in BAT of M-KO embryos, respectively (Fig. 1-S1b-c). Gene ontology (GO) analysis showed that down-regulated genes are associated with lipid and fatty acid metabolism, brown fat cell differentiation and insulin response (Fig. 1e). Notably, expression levels of insulin response genes, including *adiponectin* (*Adipoq*) and *insulin-induced gene 1* (*Insig1*), as well as lipogenesis genes such as *Slc25a1*, *Fasn*, *Scd1*, and *Agpat2*, were consistently decreased across all three replicates of M-KO mice. In contrast, genes encoding adipogenesis markers, lipogenic TFs, and lipid uptake proteins showed minimal changes (Fig. 1f-g). These results indicate that while deletion of *Hira* only moderately affects embryonic development of BAT, it reduces insulin response and lipogenesis gene expression.

### Mice with adipocyte-specific deletion of *Hira* show reduced adiponectin levels and insulin resistance under normal chow diet

**Figure 2.**
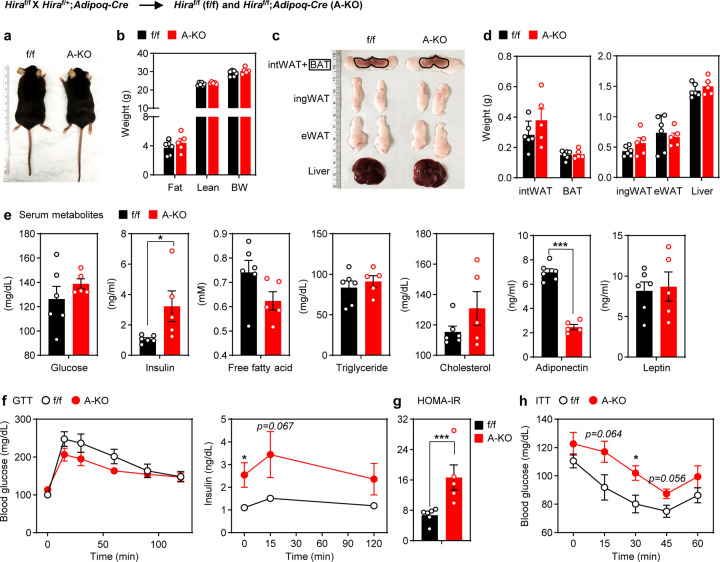
Mice with adipocyte-specific deletion of *Hira* show reduced adiponectin levels and insulin resistance under normal chow diet All data were from 23-week old *Hira*^f/f^ (f/f) and *Hira*^f/f^;*Adipoq-Cre* (A-KO) male mice fed with a normal chow diet (*n* = 5~6 per group). **a**, Representative morphology of mice. **b**, Body composition measured by MRI. **c**, Representative pictures of interscapular WAT (intWAT), BAT, inguinal WAT (ingWAT), epididymal WAT (eWAT) and liver. **d**, Average tissue weights. **e**, Levels of serum metabolites in randomly fed state. **f**, Glucose tolerance test (GTT): blood glucose (left) and plasma insulin levels (right). **g**, Insulin sensitivity was determined by HOMA-IR. **h**, Insulin tolerance test (ITT). All quantitative data for mice are presented as means ± SEM. Statistical comparison between groups was performed using Student’s *t*-test. (∗) *P* < 0.05, (∗∗) *P* < 0.01, (∗∗∗) *P* < 0.001.

To investigate the functional role of HIRA in adipose tissues, we generated adipocyte-specific *Hira* KO (*Hira*^f/f^*;Adipoq-Cre* [A-KO]) mice by crossing *Hira*^f/f^ with *Adipoq-Cre* mice. qRT-PCR confirmed the specific deletion of *Hira* in adipose tissues but not liver (Fig. 2-S1a). A-KO and f/f mice showed similar appearance, body weight, fat and lean mass (Fig. 2a-b). The weight and size of adipose tissues including interscapular white adipose tissue (intWAT), BAT, inguinal WAT (ingWAT) and epididymal WAT (eWAT) and liver were similar between f/f and A-KO mice (Fig. 2c-d). Histological analysis also revealed comparable lipid droplets in adipose tissues and liver in f/f and A-KO mice (Fig. 2-S1b).

Interestingly, serum insulin levels were significantly elevated while adiponectin levels were around 3-fold lower in A-KO compared to f/f mice (Fig. 2e). No significant changes in glucose, free fatty acid, triglyceride, cholesterol and leptin levels were observed in A-KO mice. In the glucose tolerance test (GTT), fasting blood glucose and glucose excursion curves were comparable in A-KO and controls. However, A-KO mice displayed significantly elevated fasting insulin levels and a 2-fold higher HOMA-IR (Homeostatic Model Assessment for Insulin Resistance) index, consistent with the whole-body insulin resistance (Fig. 2f-g) with glucose uptake genes remaining largely unchanged in the liver (Fig. 2-S1c). Insulin tolerance test (ITT) also revealed increased glucose levels in A-KO mice, with a significant difference at 30 min (Fig. 2h). Similar metabolic phenotype was observed in female A-KO mice tested at 21 weeks old (Fig. 2-S2). Taken together, these data indicate that HIRA is important for glucose homeostasis and that deletion of *Hira* in adipocytes causes insulin resistance in mice.

**Figure 3.**
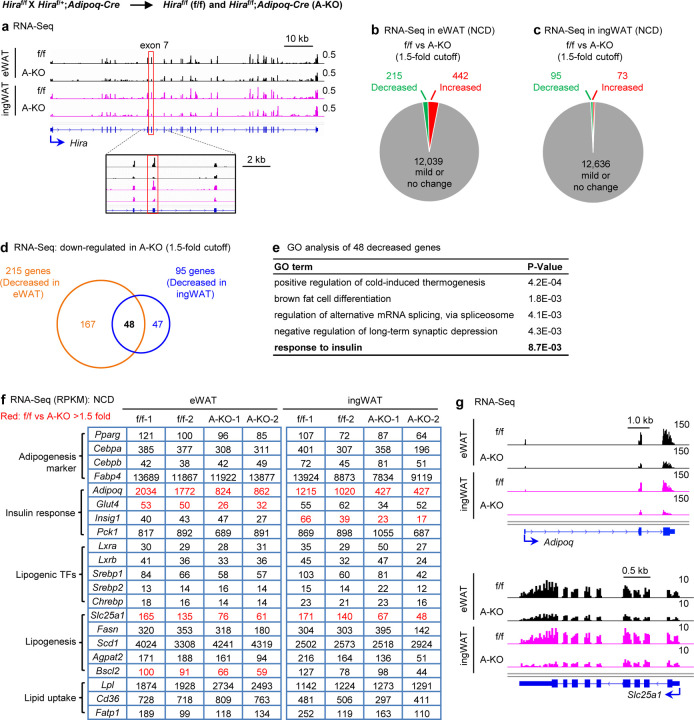
Deletion of *Hira* in adipocytes reduces adiponectin expression under normal chow diet All data were from 23-week old *Hira*^f/f^ (f/f) and *Hira*^f/f^;*Adipoq-Cre* (A-KO) male mice fed with a normal chow diet (*n* = 5~6 per group). **a**, Genome browser view of RNA-Seq data on the *Hira* gene locus. The targeted exon 7 is highlighted. **b-c**. RNA-Seq analysis of eWAT (**b**) and ingWAT (**c**). The cutoff for decreased or increased genes is 1.5-fold. **d**, Venn diagrams depicting down-regulated genes in adipose tissues of A-KO mice. **e**, GO analysis of gene groups defined in d. **f**, Expression levels of representative genes are shown in RPKM values of RNA-Seq data. **g**, RNA-Seq profiles on *Adipoq* and *Slc25a1* loci in eWAT and ingWAT. RNA-Seq analysis was done using biological replicates.

To investigate how *Hira* KO in adipocytes disrupts glucose homeostasis and contributes to insulin resistance, we performed RNA-Seq analysis in eWAT and ingWAT. We confirmed the deletion of *Hira* in both tissues (Fig. 3a). Using a 1.5-fold cutoff for differential gene expression, we identified 215 downregulated and 442 upregulated genes in eWAT, and 95 downregulated and 73 upregulated genes in ingWAT of A-KO mice compared to f/f mice (Fig. 3b-c). We found 48 genes downregulated over 1.5-fold in both eWAT and ingWAT of A-KO mice (Fig. 3d). These included *Adipoq*, which encodes adiponectin and several genes involved in lipogenesis (Fig. 3e-g). Since reduced adiponectin levels cause insulin resistance in mice^22,23^,our data suggest that HIRA may regulate insulin response/sensitivity, at least in part, by controlling *Adipoq* expression.

### Mice with adipocyte-specific deletion of *Hira* gain less fat mass during HFD-induced obesity

**Figure 4.**
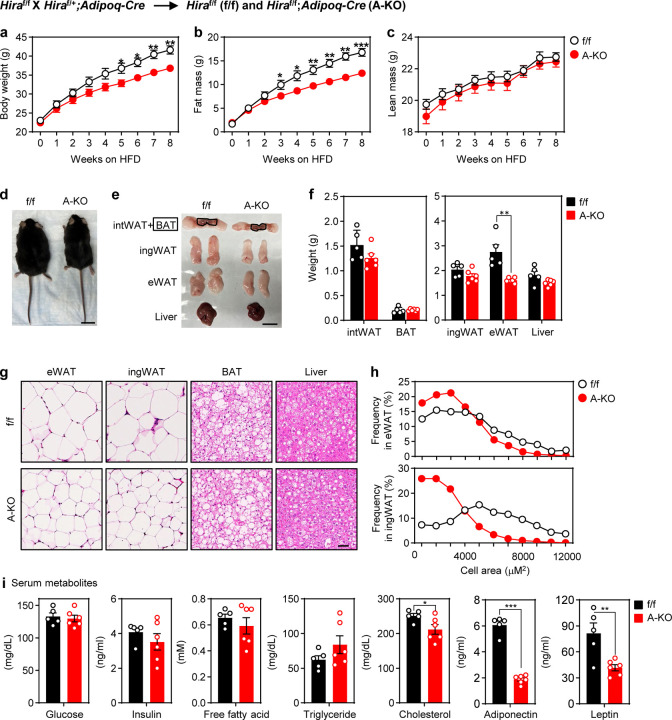
Mice with adipocyte-specific deletion of *Hira* gain less fat mass during HFD-induced obesity Male *Hira*^f/f^ (f/f) and *Hira*^f/f^;*Adipoq-Cre* (A-KO) mice (*n* = 5~6 per group) were fed with HFD from the eighth week of age. **a-c**, Total body weight (**a**), fat mass (**b**), and lean mass (**c**) were measured by MRI during HFD feeding. **d**, Representative morphology of HFD-fed mice. Scale, 2 cm. **e**, Representative pictures of intWAT, BAT, ingWAT, eWAT and liver. Scale, 2 cm. **f**, Average tissue weights. **g**, H&E staining of eWAT, ingWAT, BAT and liver. Scale bar, 50 μm. **h**, Average cell area in eWAT (upper) and ingWAT (lower). **i**, Levels of serum metabolites. All quantitative data for mice are presented as means ± SEM. Statistical comparison between groups was performed using Student’s *t*-test. (∗) *P* < 0.05, (∗∗) *P* < 0.01, (∗∗∗) *P* < 0.001.

Next, we examined the role of HIRA in diet-induced obesity. Eight-week-old f/f and A-KO mice were fed with HFD for 8 weeks. qRT-PCR confirmed the deletion of *Hira* in adipose tissues but not liver after 8 weeks on the HFD (Fig. 4-S1a). A-KO mice gained a similar amount of lean mass, but significantly less fat mass and body weight compared to f/f mice (Fig. 4a-d) with lower cumulative food intake starting at week 4 and largely unchanged energy expenditure (Fig. 4-S1b-c). Among adipose tissues examined, A-KO mice showed less gain of eWAT compared to f/f mice (Fig. 4e-f). Histological analysis revealed a substantially higher proportion of smaller adipocytes in eWAT and ingWAT of A-KO mice (Fig. 4g-h). A-KO mice exhibited comparable levels of glucose and insulin tolerance to f/f mice (Fig. 4-S1d-f). However, levels of total cholesterol, leptin and adiponectin were significantly lower in the serum of A-KO mice (Fig. 4i).

**Figure 5.**
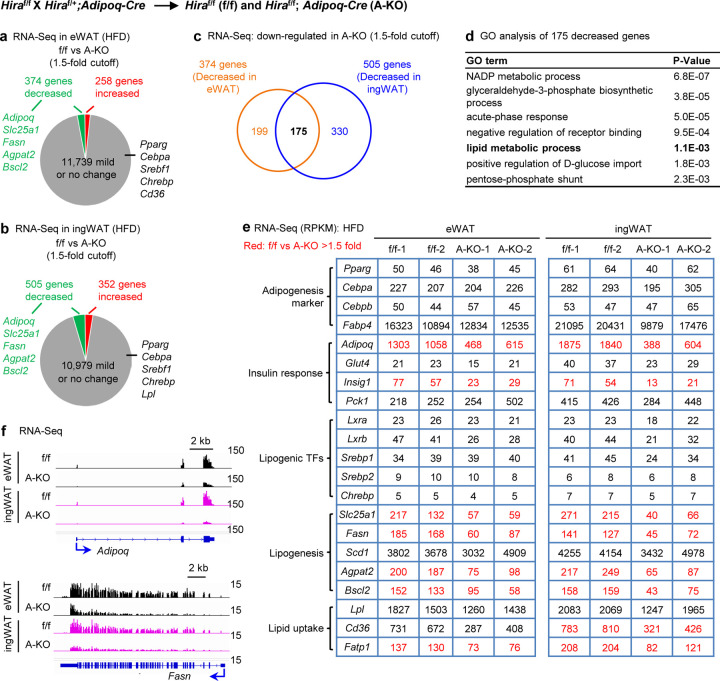
Adipocyte-specific deletion of *Hira* reduces insulin response and lipogenesis gene expression in WAT during HFD-induced obesity All data were from *Hira*^f/f^ (f/f) and *Hira*^f/f^;*Adipoq-Cre* (A-KO) mice (*n* = 5~6 per group) fed with HFD for 8 weeks from the eighth week of age. **a-b**. RNA-Seq analysis of eWAT (**a**) and ingWAT (**b**). The cutoff for decreased or increased genes is 1.5-fold. **c**, Venn diagrams depicting down-regulated genes in adipose tissues of A-KO mice. **d**, GO analysis of gene groups defined in c. **e**, Expression levels of representative genes are shown in RPKM values of RNA-Seq data. **f**, RNA-Seq profiles on *Adipoq* and *Fasn* loci in eWAT and ingWAT. RNA-Seq analysis was done using biological replicates.

We then performed RNA-Seq in eWAT and ingWAT after HFD. Using a 1.5-fold cutoff for differential gene expression, we identified 374 downregulated and 258 upregulated genes in eWAT, and 505 downregulated and 352 upregulated genes in ingWAT of A-KO mice compared to f/f mice (Fig. 5a-b). Among these, 175 genes were consistently downregulated by more than 1.5-fold in both eWAT and ingWAT of A-KO mice (Fig. 5c), showing functional enrichment in lipid metabolic process (Fig. 5d). In addition to insulin response genes such as *Adipoq* and *Insig1*, expression levels of lipogenesis genes, such as *Slc25a1*, *Fasn*, *Agpat2* and *Bscl2*, were reduced in both eWAT and ingWAT of A-KO mice, while genes encoding adipogenesis markers and lipogenic TFs remained largely unchanged (Fig. 5e-f). These results suggest that HIRA plays an important role in adipose tissue expansion during HFD-induced obesity, at least in part, by regulating insulin response and lipogenesis gene expression.

### HIRA targets insulin response and lipogenesis genes in adipocytes

**Figure 6.**
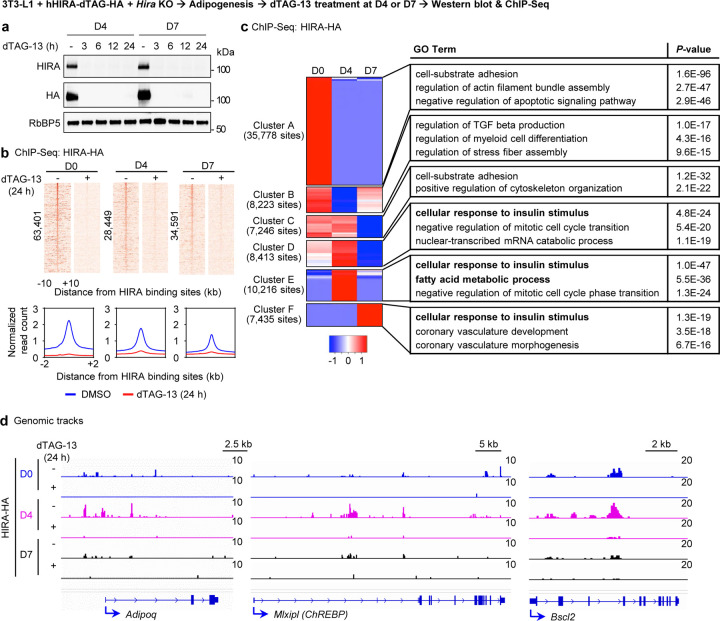
HIRA targets insulin response and lipogenesis genes in adipocytes 3T3-L1 white preadipocytes were infected with a lentiviral vector expressing human HIRA with C-terminal dTAG and HA double tags, followed by lentiviral CRISPR/Cas9-*Hira* gRNA infection to delete endogenous *Hira*. Adipogenesis assay was performed, followed by dTAG-13 treatment at D0, D4, or D7. **a**, Western blot analysis using indicated antibodies. Cells were treated with dTAG-13 for various time periods as indicated. RbBP5 was used as a loading control. **b-d**, ChIP-Seq analysis of HIRA genomic binding in the presence or absence of dTAG-13 treatment for 24 h. **b**, Heat maps (upper) and average profiles (lower) were aligned around the center of HIRA binding sites at D0 (63,401), D4 (28,449) and D7 (34,591). **c**, Heatmaps of K-means clustering depicting HIRA binding regions at D0, D4 and D7. **d**, ChIP-Seq profiles of HIRA binding and H3K27ac enrichment on *Adipoq*, *Mlxipl* (*ChREBP*) and *Bscl2* gene loci at indicated time points.

To determine whether HIRA directly regulates insulin response and lipogenesis genes in adipocytes, we introduced exogenous human HIRA with C-terminal dTAG and HA double tags into 3T3-L1 white preadipocytes and then deleted endogenous mouse *Hira* gene before inducing adipogenesis (Fig. 6-S1a). This approach enabled us to map high-confident genomic binding regions of HIRA using a ChIP-Seq quality HA antibody and dTAG13-mediated protein depletion^24^ . Western blotting and ChIP-Seq confirmed efficient depletion of HA-tagged HIRA before (day 0, D0), during (day 4, D4), and after (day 7, D7) adipogenesis (Fig. 6a-b). ChIP-Seq analysis of HA-tagged HIRA successfully identified 63,401, 28,441, and 34,591 HIRA binding regions at D0, D4 and D7 of adipogenesis, respectively. dTAG-13 treatment for 24h eliminated over 90% of HIRA-HA genomic binding. At D0, D4 and D7, 63,375 out of 63,401, 28,021 out of 28,449 and 32,400 out of 34,591 HIRA-binding regions exhibited a more than 2-fold decrease in HIRA binding, respectively (Fig. 6b). Unbiased K-means clustering analysis revealed that genes with significantly increased HIRA binding at D4 were functionally associated with “cellular response to insulin stimulus” and “fatty acid metabolic process” (Fig. 6c). Genomic tracks showed that HIRA directly binds to promoters and/or enhancers of *Adipoq*, *Mlxipl* encoding the lipogenic TF ChREBP, as well as lipogenesis genes such as *Bscl2* (Fig. 6d). These results indicate that HIRA directly targets insulin response and lipogenesis genes in differentiating adipocytes.

### Acute depletion of HIRA impairs transcription elongation on target genes in adipocytes

**Figure 7.**
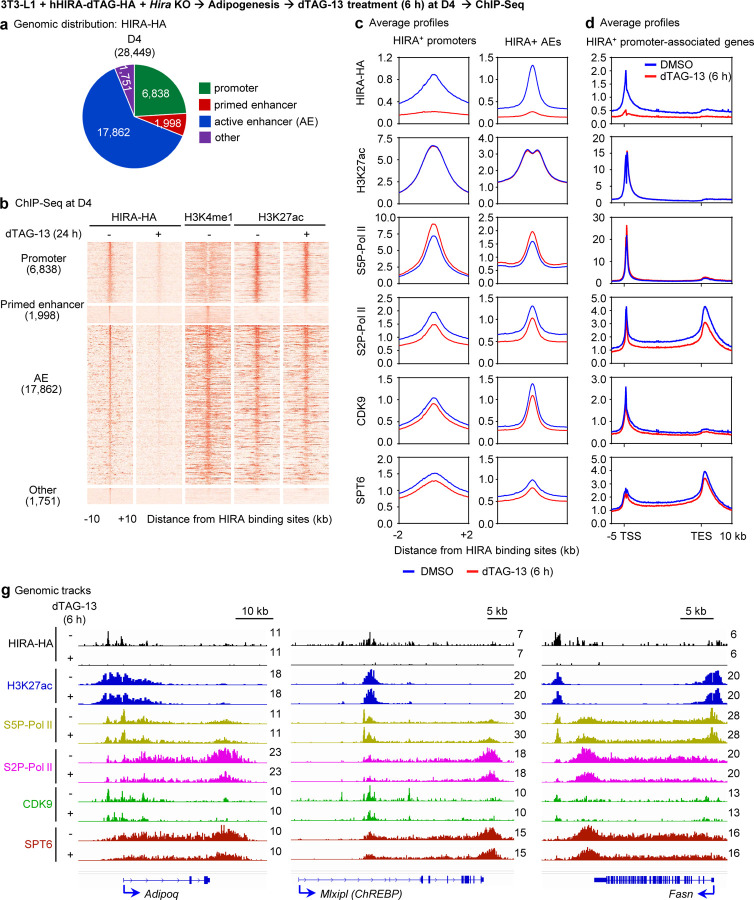
Acute depletion of HIRA impairs transcription elongation on target genes in adipocytes 3T3-L1 white preadipocytes were infected with a lentiviral vector expressing human HIRA with C-terminal dTAG and HA double tags, followed by lentiviral CRISPR/Cas9-*Hira* gRNA to delete endogenous *Hira*. At D4 of adipogenesis, cells were treated with dTAG-13 for 6h. **a**, Pie charts depicting the genomic distribution of HIRA binding regions at D4. **b**, Heat maps were aligned around the center of HIRA binding sites on promoters (6,838), primed enhancers (1,998), active enhancers (AEs, 17,862) and other regions (1,751) at D4. **c**, Average binding profiles of HIRA-HA, H3K27ac, S5P-Pol II, S2P-Pol II, CDK9 and SPT6 around the center of HIRA^+^ promoters and AEs. Normalized read counts are shown. **d**, Profiles of HIRA-HA, H3K27ac, S5P-Pol II, S2P-Pol II, CDK9 and SPT6 on gene bodies of HIRA^+^ promoter-associated genes. **g**, ChIP–Seq profiles of HIRA-HA, H3K27ac, S5P-Pol II, S2P-Pol II, CDK9 and SPT6 were displayed on *Adipoq*, *Mlxipl (ChREBP)* and *Fasn* loci.

We further characterized the genomic distribution of HIRA in adipocytes. At D4 of adipogenesis, HIRA preferentially binds promoters and enhancers. Among the 28,449 HIRA binding regions at D4 of adipogenesis, 17,862 (62.8%) were located on active enhancers (AEs), while 6,838 (24%) were located on promoters (Fig. 7a-b). To minimize potential secondary effects caused by permanent *Hira* gene deletion, we utilized 6-hour dTAG-13 treatment to acutely deplete HIRA protein at the genomic level at D4 of adipogenesis. Interestingly, HIRA depletion did not alter the enrichment of the AE mark H3K27ac (Fig. 7c), suggesting that HIRA is dispensable for maintaining AEs. However, acute HIRA depletion led to decreased levels of serine 2 phosphorylated (S2P) elongating RNA polymerase II (S2P-Pol II) and transcription elongation factors, including CDK9 and SPT6, while increasing serine 5 phosphorylated (S5P) initiating Pol II levels (S5P-Pol II) on HIRA^+^ promoters, AEs, and gene bodies (Fig. 7c-d). Consistently, decreased levels of S2P-Pol II, CDK9, and SPT6 were observed on enhancers and promoters of insulin response gene *Adipoq*, lipogenic TF *ChREBP* and lipogenesis gene *Fasn* (Fig. 7e). Together, our data suggest that HIRA regulates insulin response and lipogenesis gene expression by facilitating Pol II elongation.

## Discussion

By tissue-specific gene KO in mice, we identify the histone chaperone HIRA as a novel epigenomic regulator of insulin sensitivity and obesity-associated adipose tissue expansion. Deletion of *Hira* reduces insulin response and lipogenesis gene expression in adipocytes in mice. By ChIP-Seq, we determine genomic binding sites of HIRA and show that HIRA targets promoters and enhancers of insulin response and lipogenesis genes in adipocytes. Finally, acute HIRA protein degradation in adipocytes reveals that HIRA is required for transcription elongation on target genes, providing a novel mechanism by which HIRA regulates transcription.

By crossing *Hira*^f/f^ with *Myf5-Cre* mice, we observed that *Hira* KO had a mild effect on the size of embryonic BAT and the expression of adipogenesis master regulators PPARγ and C/EBPα (Fig. 1d, f). However, *Hira* KO mice displayed a decreased muscle mass at the E18.5 stage and were unable to survive to weaning age and *Hira* KO cells failed to undergo myogenesis. These findings suggest that HIRA is required for muscle development, which is consistent with previous findings^21,25^. In adult mice, *Pax7*-Cre mediated deletion of *Hira* in satellite cells leads to defective muscle regeneration following injury^21^. Additionally, mice with skeletal muscle-specific deletion of *Hira* via *Myf6-Cre* develop hypertrophy^26^. Together, these observations suggest that HIRA plays important functional roles at various stages of muscle development.

Under normal chow diet, both male and female A-KO mice maintain normal body and adipose tissue weights but show significantly decreased serum levels of the insulin-sensitizing adipokine adiponectin, which may contribute to the observed insulin resistance phenotype. Reduced expression of lipogenesis genes such as *Slc25a1* in these mice may also contribute to insulin resistance, because insulin resistance is associated with reduced lipogenesis enzyme expression in WAT^6^. Under HFD, the reduced fat mass gain and smaller adipocytes observed in A-KO mice can be partially explained by the decreased expression of lipogenesis genes such as *Fasn*. Consistently, it has been reported that adipocyte-specific *Fasn* KO mice maintain normal body weight under normal chow diet but display reduced fat mass and smaller eWAT under HFD^27^. Besides this, the reduced cumulative food intake likely contributes to the decreased fat mass and body weight in A-KO mice, which could also be related to low adiponectin levels since adiponectin-deficient mice showed decreased food intake and exhibited resistance to HFD-induced obesity^28^. Furthermore, despite decreased adiponectin levels, *Hira* KO mice did not show more severe insulin resistance compared to controls under HFD. This could be due to the concurrent approximately 50% reduced leptin levels observed in *Hira* KO mice (Fig. 4i), since a 40%–50% reduction in circulating leptin levels has been reported to enhance insulin sensitivity in mice under HFD^29^.

Using adipogenesis as a model system, we examined HIRA genomic localization in cell differentiation. We used a ChIP-Seq quality HA antibody to detect the exogenous HA tagged HIRA because the available HIRA antibody worked for western blot but failed in ChIP-Seq. In differentiating adipocytes (D4), HIRA predominantly binds to promoters, primed enhancers, and AEs, consistent with previous findings from HeLa cells^16^. HIRA binding sites highly associate with insulin response and lipogenesis genes, indicating a direct regulatory role of HIRA. Since adipogenic and/or lipogenic TFs PPARγ, C/EBPα, SREBP1 and ChREBP directly bind insulin response gene *Adipoq* (Fig. 6-S1b) and lipogenesis genes and regulate their expression^8,30^, future studies will be needed to find out whether any of these TFs are responsible for recruiting HIRA to these target genes.

HIRA is enriched on AEs, but acute depletion of HIRA has little effect on H3K27ac levels (Fig. 7c), suggesting that HIRA works downstream of enhancer activation by H3K27 acetyltransferases CBP and p300^31^. It remains to be investigated whether HIRA is recruited by CBP/p300 or downstream chromatin factors enriched on AEs, such as BRD4 and the lipogenesis coactivator MED1^8,32^. By acute depletion of HIRA, we demonstrated a direct and specific role for HIRA in transcription elongation, rather than initiation, particularly on insulin response and lipogenesis-associated genes. Future studies investigating the potential interactions between HIRA and elongation factors, as well as the impact of HIRA depletion on H3.3 deposition, may provide deeper insights into how HIRA regulates transcription elongation.

## Materials and Methods

### Plasmids, antibodies, and chemicals

Lentiviral plasmid pLV-EF1a-IRES-hygro–hHIRA-dTAG-HA was generated by following steps: (1) dTAG-HA (FKBP_F36V-HA) fragment (Addgene #91796) was C-terminally subcloned into lentiviral vector pLV-EF1a-IRES-hygro (Addgene #85134) to generate pLV-EF1a-IRES-hygro-dTAG-HA. (2) Human *HIRA* was PCR-amplified using cDNA from 293T cells as templates and was subcloned into pLV-EF1a-IRES-hygro-dTAG-HA. (3) Q5 Site-Directed Mutagenesis Kit (NEB #E0554S) was used to mutate gRNA sequence in the plasmid. Plasmid was confirmed by DNA sequencing. Anti-HIRA antibody (04–1488) was from Sigma. Anti-HA (3724S) for western blot was from Cell Signaling Technology. Anti-HA (13–2010) for ChIP-Seq was from Epicypher. Anti-H3K27ac (ab4729) was from Abcam. Anti-RbBP5 (A300–109A) was from Bethyl Laboratories. Anti-S5P-Pol II (13523), anti-S2P-Pol II (13499S), anti-CDK9 (2316T) and anti-SPT6 (15616) were from Cell Signaling Technology. 500nM dTAG-13 (Biotechne #6605) was used for depletion of hHIRA-dTAG-HA.

### Generation of mouse strains

*Hira*^f/f^ mice^20^ were crossed with *Myf5-Cre* (Jackson 007893) or *Adipoq-Cre* (Jackson 028020) to generate *Hira*^f/f^;*Myf5-Cre* or *Hira*^f/f^;*Adipoq-Cre*, respectively. For genotyping *Hira* alleles, PCR was performed using following primers: 5′-TGCTAAAGAAAAACTAGCCGAG-3′ and 5′-TGTGTTTGGTACCCCACAAC-3′. PCR amplified 190 bp from the wild-type and 300 bp from the floxed allele.

All mouse experiments were performed in accordance with the NIH Guide for the Care and Use of Laboratory Animals and approved by the Animal Care and Use Committee of the National Institute of Diabetes and Digestive and Kidney Diseases, NIH.

### Metabolic studies

For determination of serum metabolites, blood was collected from the tail vein of mice fed with standard laboratory mouse chow (13.6% calories from fat, 60% calories from carbohydrate, 26.4% calories from protein; LabDiet 5018) or high-fat diet (60% calories from fat, 20% calories from carbohydrate, 20% calories from protein; Research Diets D12492). Serum was obtained by centrifuging blood samples at 12,000*g* for 4 min at room temperature. Serum insulin and leptin concentrations were measured by an ELISA kit from CrystalChem and R&D Systems, respectively. Serum concentration of free fatty acid (Fujifilm), free glycerol (Sigma), total triglyceride (Pointe Scientific Inc.), and total cholesterol (Pointe Scientific Inc.) were measured using the indicated calorimetric assay. For glucose tolerance tests (GTT), mice were fasted overnight for 16 h. For insulin tolerance tests (ITT), mice were fasted for 4 h. Glucose (1 g/kg) or human insulin (0.75 U/kg Humulin, Eli Lilly), respectively, were administered intraperitoneally (i.p.), and blood was collected from the tail vein at specific time points. For both tests, blood glucose levels were determined using a portable glucometer (Contour Glucometer, Bayer). HOMA-IR (Homeostatic model assessment for insulin resistance) index was calculated as previously described^33^.

Body composition was measured with the EchoMRI 3-in-1 analyzer (Echo Medical Systems). In singly housed mice fed with HFD, body composition and food intake were measured weekly over the course of 8 weeks, and weekly energy expenditure was calculated by energy balance technique^34^.

### Generation of 3T3-L1 white preadipocytes expressing HIRA-dTAG-HA and adipogenesis

3T3L1 white preadipocytes were infected with lentiviral hHIRA-dTAG-HA, followed by lentiviral CRISPR/Cas9 *Hira* gRNA infection. gRNA sequence to knock out *Hira*: TGTGTGCGGTGGTCAAACAG.

For the adipogenesis assay, cells were plated in growth medium (DMEM plus 10% bovine serum 4 days before the induction and were induced with 10 μg/mL insulin, 0.5 mM IBMX and 1 μM DEX in fetal bovine serum (FBS) medium (DMEM plus 10% FBS) for 2 days. After 2 days of induction, the culture medium was changed to DMEM supplemented with FBS and insulin only^35^.

### Western blot and qRT-PCR

Western blot of nuclear extracts was done as described^8^. Total RNA was extracted using TRIzol (Invitrogen) and reverse-transcribed using a ProtoScript II first strand cDNA synthesis kit (NEB), following the manufacturer’s protocols. qRT-PCR SYBR primers are shown in Supplementary Table 1.

### RNA-Seq library preparation

Total RNA (1 μg) was subjected to the NEBNext poly(A) mRNA magnetic isolation module (NEB) to isolate mRNA and proceeded directly to double-stranded cDNA synthesis. Library construction was done using the NEBNext Ultra II RNA library preparation kit for Illumina (NEB) following the manufacturer’s protocol.

### ChIP-Seq library preparation

ChIP-Seq was performed as described in detail previously^36^. ChIP-Seq was done in the presence of *Drosophila* spike-in chromatin and antibody following the manufacturer’s protocol (Active Motif). ChIP-Seq library construction was done using a NEBNext Ultra II DNA library preparation kit for Illumina (NEB) following the manufacturer’s protocol. Sequencing libraries were analyzed with Qubit and pooled and sequenced on a NovaSeq 6000, NovaSeq X, or NextSeq 2000.

### Computational analysis

#### RNA-Seq data analysis

Sequencing data were aligned to the mouse mm9 reference genome using the STAR software^37^ with default parameters. Aligned reads on exons were calculated to determine the reads per kilobase per million (RPKM) as a measurement of gene expression. Genes with RPKM values exceeding 5 were considered as expressed. Differentially expressed genes were identified using DESeq2^38^ in R (v3.5.3), applying a threshold of 1.5-fold change and a *P*-value < 0.01. To identify the functional significance of differentially expressed genes, Gene ontology (GO) analysis was performed using the DAVID tool^39^ (https://davidbioinformatics.nih.gov/home.jsp).

#### ChIP-Seq peak calling

Sequencing data were aligned to the mouse mm9 reference genome using Bowtie2. For the identification of ChIP-Seq enriched regions, SICER (version 2)^40^ was used. For HIRA-HA and SPT6, a window size of 50 bp, gap size of 50 bp, and a false discovery rate of 1E-10 were used. For S5P-Pol II, S2P-Pol II, and CDK9, a window size of 50 bp, gap size of 50 bp, and a false discovery rate of 1E-3 were used. For H3K27ac, a window size of 200 bp, gap size of 200 bp, and a false discovery rate of 1E-3 were used. The effective genome fraction of 0.8 was applied to all ChIP-Seq data.

#### Normalization of ChIP-Seq data

To assess the Spike-in chromatin incorporated into the ChIP reaction, sequencing data were aligned to the drosophila dm6 reference genome using Bowtie2. For data normalization, the sample with the fewest drosophila reads was first identified. Then, the normalization factor was determined by dividing the drosophila reads of the sample with the lowest count by those of other samples. After calculation, each sample’s reads were scaled by multiplying them with their respective normalization factor to generate heatmap matrices or profiles.

## Supplementary Material

Supplement 1

Supplement 2

## Data Availability

RNA-Seq and ChIP-Seq data sets generated in the paper have been deposited in NCBI Gene Expression Omnibus under accession number GSE288013.

## Bullet points

- HIRA depletion reduces expression of insulin response and lipogenesis genes in adipose tissue
- HIRA depletion alleviates adipose tissue expansion during high-fat diet-induced obesity
- HIRA targets insulin response and lipogenesis genes in adipocytes
- Acute depletion of HIRA impairs transcription elongation on target genes
