# Supplementary material for: Histone chaperone HIRA facilitates transcription elongation to regulate insulin sensitivity and obesity-associated adipose expansion
Source: bioRxiv. 2025 Mar 25:2025.03.21.644577. Preprint. [Version 1] doi: 10.1101/2025.03.21.644577 (PMC11974756; doi:10.1101/2025.03.21.644577)
Supplement: Supplement 1 [file media-1.pdf]

Supplementary Table 1: qRT-PCR primers

Gene	Forward primer 5'-3'	Reverse primer 5'-3'
18s	AGTCCCTGCCCTTTGTACACA	CGATCCGAGGGCCTCACTA
Hira	TTGACTTGGGATCCCGTTGG	AGTCTCTAGCTGCCAGTCCA
Glut9	TTGCTTTAGCTTCCCTGATGTG	GAGAGGTTGTACCCGTAGAGG
Glut2	TCAGAAGACAAGATCACCGGA	GCTGGTGTGACTGTAAGTGGG
Glut8	AGCAGCGTCATGGAGATGC	ACAACGGTCAGTGTGAATAGGA
Glut1	CAGTTCGGCTATAACACTGGTG	GCCCCCGACAGAGAAGATG
