# Supplementary material for: Histone chaperone HIRA facilitates transcription elongation to regulate insulin sensitivity and obesity-associated adipose expansion
Source: bioRxiv. 2025 Mar 25:2025.03.21.644577. Preprint. [Version 1] doi: 10.1101/2025.03.21.644577 (PMC11974756; doi:10.1101/2025.03.21.644577)
Supplement: Supplement 2 [file NIHPP2025.03.21.644577v1-supplement-2.pdf]

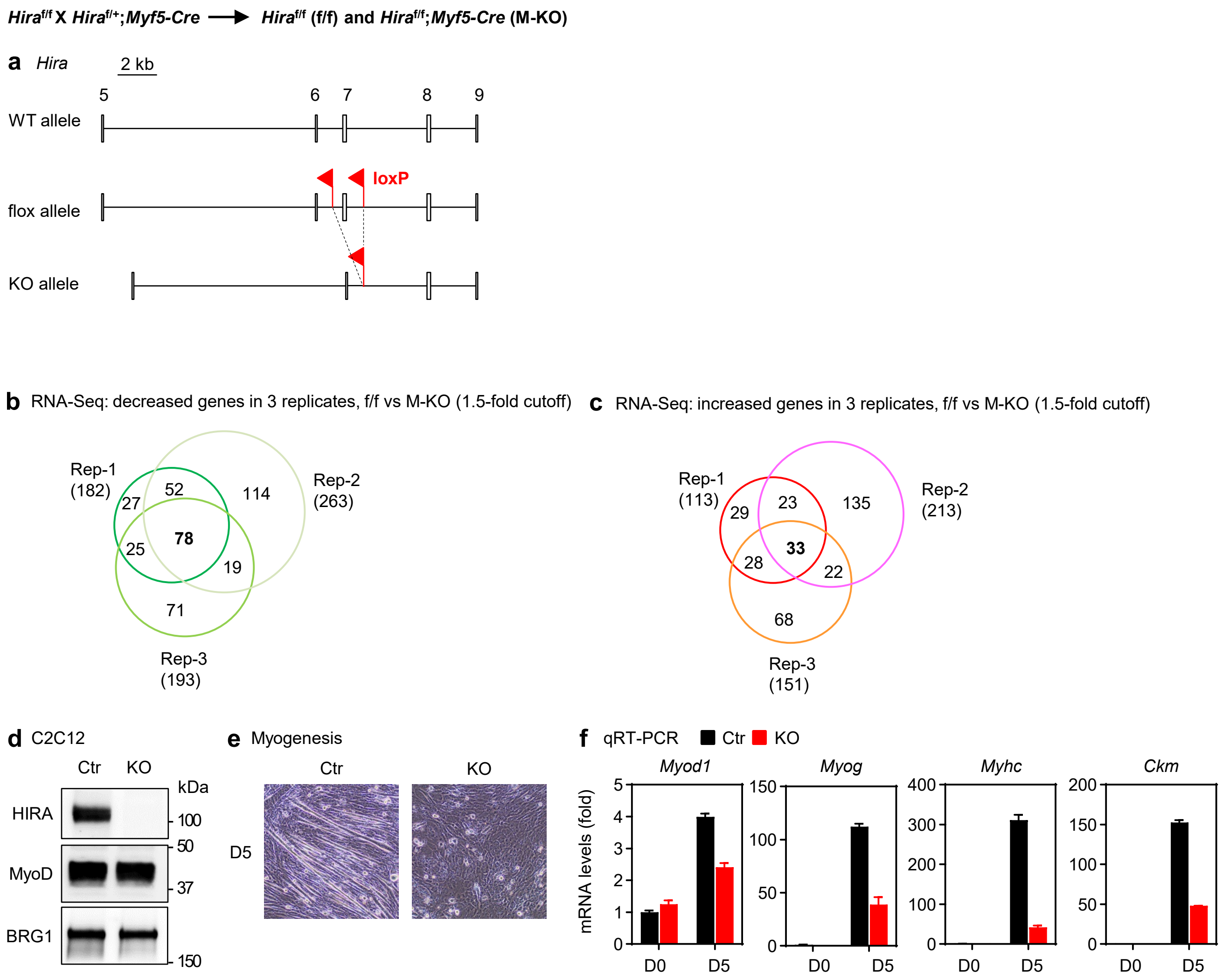

Figure 1-S1. Supporting data for Figure 1.

a, Schematics of wild-type (WT) allele, conditional KO (flox) allele, and KO allele of *Hira*$^{f/f}$ mice. In the flox allele, exon 7 was flanked by two loxP sites. **b-c**, Venn diagrams showing RNA-Seq analysis of BAT at E18.5. The cutoff for decreased (**b**) or increased (**c**) genes in *Hira*$^{f/f}$;*Myf5-Cre* (M-KO) compared with *Hira*$^{f/f}$ (f/f) in each of three replicates is 1.5-fold. The RPKM threshold is set to >5 for genes included in the differential expression analysis. **d-f**, C2C12 myoblasts were infected with a lentiviral CRISPR/Cas9-*Hira* gRNA to delete endogenous *Hira*, followed by myogenesis assay. **d**, Western blot analysis using indicated antibodies. **e**, 5 days after induction of differentiation, cell morphologies were observed under microscope. **f**, qRT-PCR analysis of myogenic gene expression before or after differentiation at day 0 (D0) or day 5 (D5), respectively.

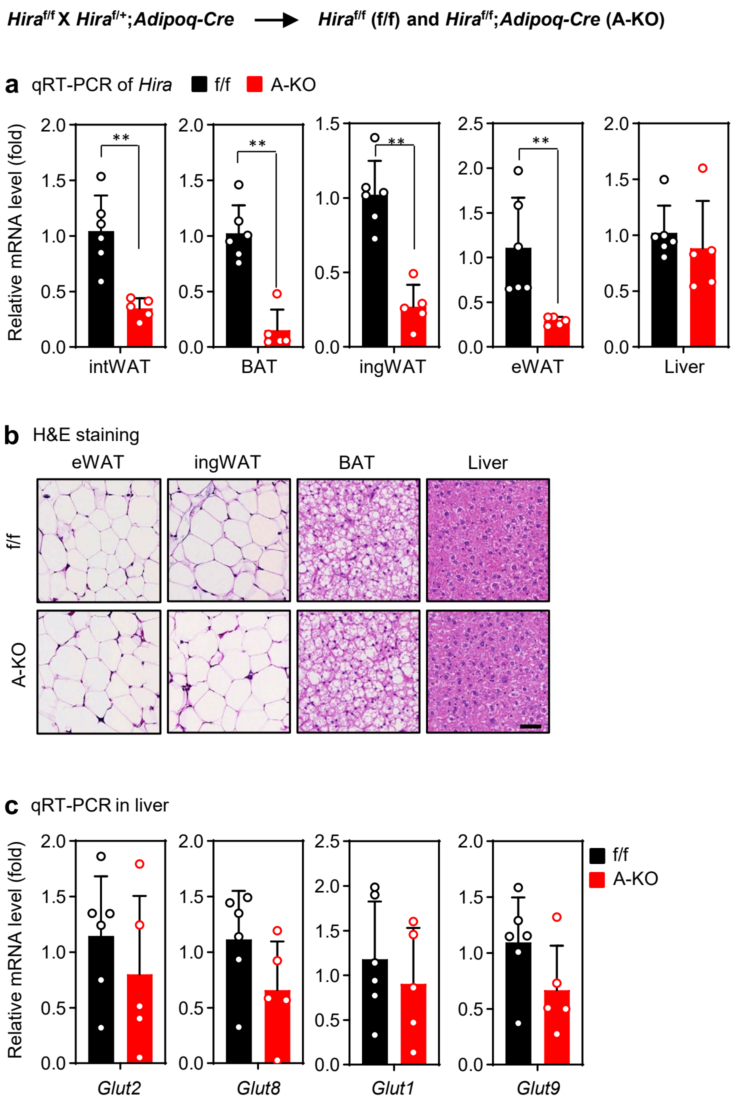

Figure 2-S1. Characterization of mice with adipocyte-specific deletion of *Hira* under normal chow diet

All data were from 23-week old *Hira*$^{f/f}$ (f/f) and *Hira*$^{f/f}$;*Adipoq-Cre* (A-KO) male mice fed with normal chow diet (n = 5~6 per group). **a**, qRT-PCR analysis of *Hira* mRNA levels. **b**, H&E staining of ingWAT, eWAT, BAT and liver. Scale bar, 50 µm. **c**, qRT-PCR analysis of glucose uptake genes in the liver. All quantitative data for mice are presented as means ± SEM. Statistical comparison between groups was performed using Student's t-test. (*) $P < 0.05$, (**) $P < 0.01$, (***) $P < 0.001$.

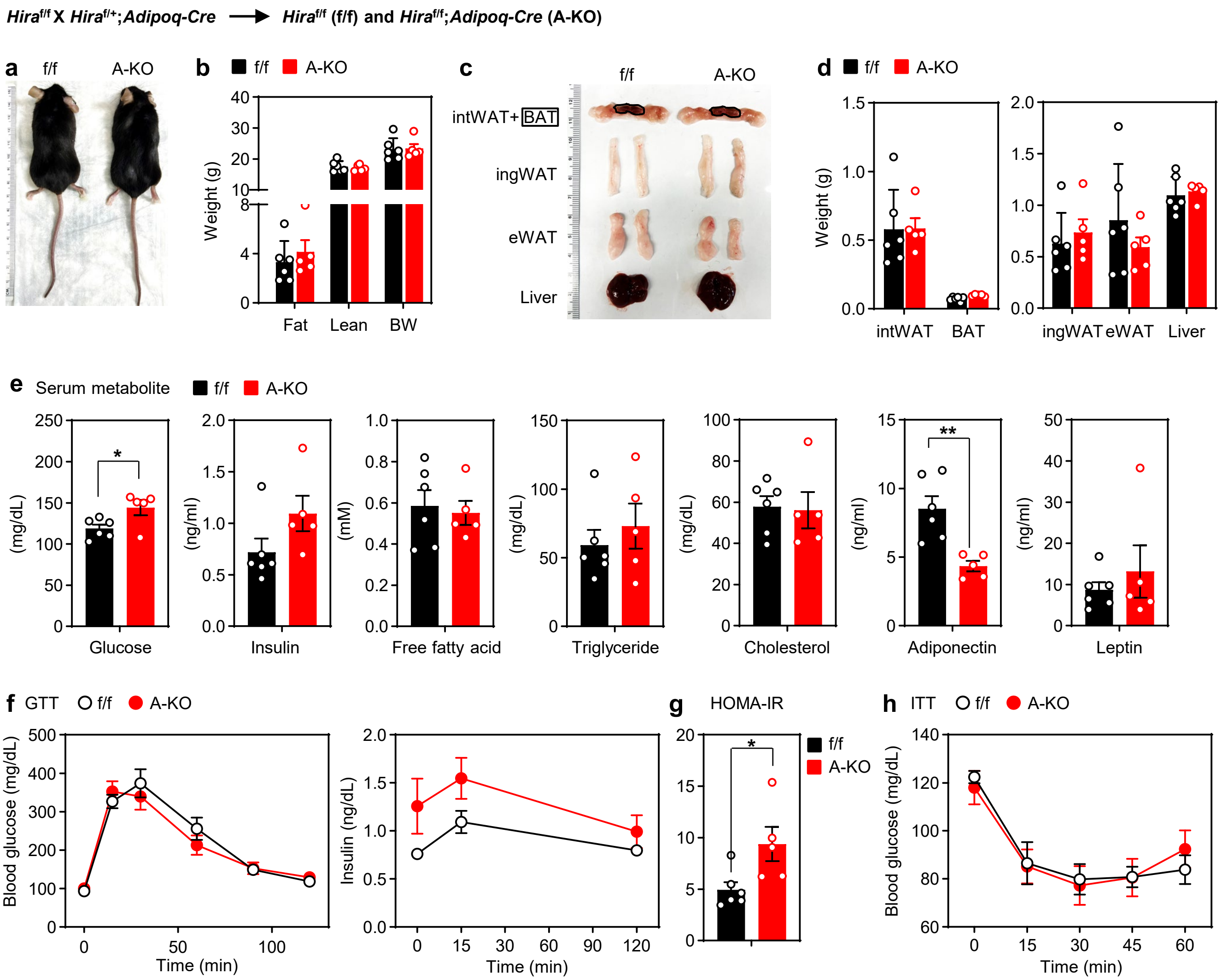

Figure 2-S2. Female mice with adipocyte-specific deletion of *Hira* show insulin resistance under normal chow diet

All data were from 21-week old *Hira*$^{f/f}$ (f/f) and *Hira*$^{f/f}$;*Adipoq-Cre* (A-KO) female mice fed with a normal chow diet (n = 5~6 per group). **a**, Representative morphology of mice. **b**, Body composition measured by MRI. **c**, Representative pictures of intWAT, BAT, ingWAT, eWAT and liver. **d**, Average tissue weights. **e**, Levels of serum metabolites. **f**, GTT (left panel) and plasma insulin levels (right panel). **g**, Insulin resistance was determined by HOMA-IR. **h**, Insulin tolerance test (ITT). All quantitative data for mice are presented as means ± SEM. Statistical comparison between groups was performed using Student's t-test. (∗) $P < 0.05$, (∗∗) $P < 0.01$, (∗∗∗) $P < 0.001$.

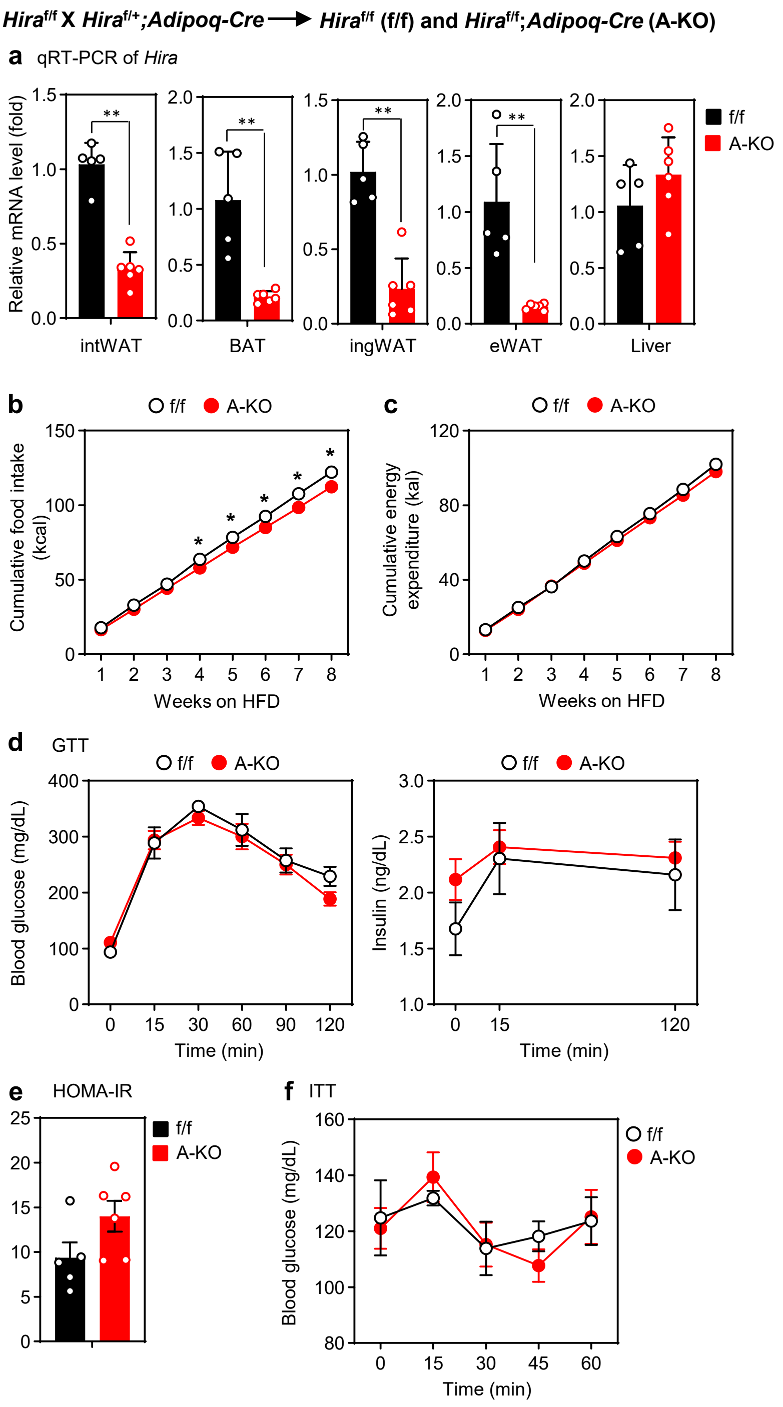

Figure 4-S1. Characterization of mice with adipocyte-specific deletion of *Hira* during HFD-induced obesity

Male *Hira*$^{f/f}$ (f/f) and *Hira*$^{f/f}$;AdipoqCre (A-KO) mice (*n* = 5~6 per group) were fed with HFD from the eighth week of age. **a**, qRT-PCR analysis of *Hira* mRNA levels in different tissues. **b-c**, cumulative food intake (**b**) and cumulative energy expenditure (**c**). **d**, GTT: blood glucose (left) and plasma insulin levels (right). **e**, Insulin sensitivity was determined by HOMA-IR. **f**, ITT. All quantitative data for mice are presented as means ± SEM. Statistical comparison between groups was performed using Student's *t*-test. (∗) $P < 0.05$, (∗∗) $P < 0.01$, (∗∗∗) $P < 0.001$.

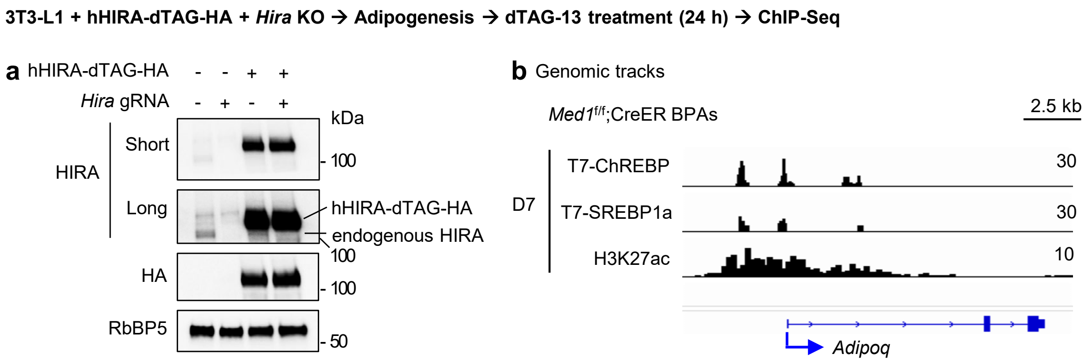

Figure 6-S1. Supporting data for Figure 6

a, 3T3-L1 white preadipocytes were infected with a lentiviral vector expressing human HIRA with C-terminal dTAG and HA double tags, followed by lentiviral CRISPR/Cas9-*Hira* gRNA to delete endogenous *Hira*. Western blot analyses in undifferentiated cells using antibodies indicated on the left. **b**, ChIP-Seq profiles of T7-ChREBP, T7-SREBP1a and H3K27ac on the *Adipoq* gene locus in adipocytes at D7. Data are from GSE160605[8].
